# Serum Hepcidin as a Biomarker of Subclinical Atherosclerosis in Peritoneal Dialysis: A Cross-Sectional Study

**DOI:** 10.3390/jcm14227905

**Published:** 2025-11-07

**Authors:** Emina Kostić, Zorica Dimitrijević, Branislav Apostolović, Karolina Paunović, Branka Mitić

**Affiliations:** 1 Clinic for Nephrology, Clinical Center Niš, 18000 Niš, Serbia; zorikad@yahoo.com (Z.D.); bane.apostolovic@gmail.com (B.A.); paunovickarolina@gmail.com (K.P.); miticdrbranka@gmail.com (B.M.); 2 Faculty of Medicine, University of Niš, 18000 Niš, Serbia

**Keywords:** hepcidin, carotid intima media thickness, cardiovascular disease, peritoneal dialysis

## Abstract

**Background**: Cardiovascular disease (CVD) is the leading cause of mortality in peritoneal dialysis (PD) patients, with traditional risk factors failing to fully explain the accelerated atherosclerosis observed in this group. Hepcidin, a major regulator of iron metabolism and inflammation, has emerged as a potential contributor to vascular remodeling. **Methods**: We conducted a cross-sectional study of 82 PD patients to assess the relationship between serum hepcidin levels and carotid intima–media thickness (CIMT), a surrogate marker of subclinical atherosclerosis. Clinical, biochemical, and dialysis-related data were collected. Patients were stratified into tertiles by hepcidin levels, and correlation, regression, and ROC analyses were performed. **Results**: Serum hepcidin levels showed a strong positive correlation with CIMT (ρ = 0.788, *p* < 0.001). In multivariate linear regression, hepcidin (β = 0.0057, *p* = 0.012) and dialysis duration (β = 0.0018, *p* = 0.015) remained independent predictors of CIMT. ROC analysis demonstrated excellent discriminative ability of hepcidin for elevated CIMT (AUC = 0.922), which improved further with the inclusion of dialysis duration (AUC = 0.952). **Conclusions**: Serum hepcidin is a strong, independent predictor of subclinical atherosclerosis in PD patients. These findings suggest that iron dysregulation and inflammation may play a more prominent role than traditional cardiovascular risk factors in this population. Hepcidin may serve as a valuable biomarker for early vascular risk stratification and a potential therapeutic target.

## 1. Introduction

Cardiovascular (CV) complications are the most common cause of death in patients on peritoneal dialysis (PD). Data from the United States show that in 2020, CV disease (CVD) was attributable to 52.7% of mortality among these patients [1]. Atherosclerosis is a key pathological process underlying CVD in patients on dialysis. It begins at an earlier age, progresses more rapidly, and is referred to as “accelerated atherosclerosis” [2]. Traditional risk factors do not fully explain the excessive atherosclerotic burden in these patients, and a number of novel risk factors attributable to kidney disease (anemia, calcium–phosphate complex, inflammation, oxidative stress) [3] and dialysis (bacteremia, hyperglycemia, and fluid overload) [4] are thought to have significant importance.

Among them, hepcidin, a liver-derived peptide hormone and key regulator of iron metabolism, has emerged as a potential contributor to vascular injury [5]. Hepcidin plays a crucial role in maintaining iron homeostasis by inhibiting iron absorption in enterocytes and blocking the release of iron recycled from erythrophagocytosis, limiting its bioavailability [6]. The secretion of hepcidin is upregulated by increased iron stores and inflammation, whereas it is reduced in response to iron deprivation, hypoxia, and enhanced erythropoiesis [7].

In recent years, the interaction between hepcidin and fibroblast growth factor 23 (FGF23) has attracted increasing attention. This phosphaturic hormone, which contributes to the regulation of mineral metabolism, also plays a role in the pathophysiology of anemia and inflammation [8,9]. FGF23 levels are elevated in cases of iron deficiency and anemia in both animal and human models [10,11]. An experimental study demonstrated that this hormone influences hepcidin levels in a dose-dependent manner, with low concentrations of FGF23 increasing hepcidin levels and high concentrations decreasing them [12].

It has been hypothesized that iron retention in vascular macrophages, caused by hepcidin, may induce and promote the transformation of these cells into foam cells [13], stimulating the release of chemokines implicated in the initiation and progression of atherosclerosis, inflammation, and fibroproliferative processes that change the arterial wall structure [14]. The role of hepcidin in atherosclerosis is particularly important in patients on dialysis since its expression is upregulated due to impaired renal excretion, chronic inflammatory status, iron administration, erythropoietin therapy, and inadequate dialysis [15]. Although FGF23 has also been associated with CVD both in the general population and in the chronic kidney disease (CKD) population [16,17], whether this hormone contributes to the progression of atherosclerosis is still a matter of research.

The relationship between hepcidin levels and atherosclerosis in patients on PD who are affected by altered metabolic and inflammatory states is still under research. Clarifying this relationship is critical to understanding the mechanisms underlying CVD risk in this population and identifying potential therapeutic targets.

In this study, we aimed to investigate the association between hepcidin levels and carotid intima–media thickness (CIMT) in a cohort of patients on PD. By analyzing baseline clinical, biochemical, and vascular data, we sought to determine whether hepcidin levels influence vascular remodeling and also identify other predictors of CIMT. Stratifying patients by hepcidin levels allowed us to evaluate the relationship between hepcidin and CIMT across subgroups. This approach offers novel insights into the linkage between hepcidin, iron metabolism, and vascular health in PD patients, paving the way for potential interventions to mitigate CVD risk in this population.

## 2. Materials and Methods

We performed a cross-sectional study on patients who underwent peritoneal dialysis (PD) at the Clinic for Nephrology, Clinical Center Nis, Serbia. The inclusion criteria for this study were patients with CKD over the age of 18 years who were treated with PD for at least six months. Exclusion criteria were clinical presentations of CV complications at the start of the study, the presence of acute infection and malignancy, active liver disease, hematological disorders, and previous renal transplantation. All participants were included after providing informed consent. The study was approved by the Ethics Committee of the Clinical Center Nis in March 2019, No 7839/11.

Baseline demographic and clinical data, dialysis vintage, comorbidities, and anemia medication were collected from medical records and interviews during outpatient visits.

Blood pressure (BP) measurements were taken in a sitting position after a 5 min resting period. The average of 3 measurements was used. Subjects with BP greater than 140/90 mm Hg and/or those on antihypertensive medications were considered hypertensive.

Diabetes was defined by the current guidelines [18] or by the use of insulin or oral hypoglycemic agents.

Anemia was defined as a hemoglobin concentration < 12.0 g/dL in both males and females. Transferrin saturation (TSAT) was calculated as Fe∙100/TIBC. Following the KDIGO guidelines [19], anemia treatment comprising erythropoiesis-stimulating agents (ESAs) and, if needed, iron therapy.

The patient’s height and weight were measured after draining the dialysis fluid, and body mass index (BMI) was calculated as weight/height^2^, expressed in kg/m^2^.

The patients’ smoking habits were recorded. Current and former smokers were considered to have a positive cigarette smoking history.

Blood samples were collected after 12 h of fasting. Serum biochemical parameters were measured using a computerized auto-analyzer (Dimension RxL Max Integrated Chemistry System, Siemens, Munich, Germany). Ferritin was measured using a commercial test on an immunoassay analyzer (Roche e411 Chemistry Analyzer, Roche, Basel, Switzerland).

Hepcidin was quantitatively determined by the standard ELISA method, using commercial ELISA tests on the Multiscan Ascent 96/384 Plate Reader, Thermo Fischer Scientific, Waltham, MA, USA. Serum samples were stored at −80 °C until assayed.

CIMT was measured by a single, well-trained physician who was blinded to the patient’s medical history and laboratory data. Measurements were performed on a high-resolution B-mode ultrasound system (TUS A500, Toshiba, Tokyo, Japan), with a 7.5–11 MHz transducer, using a semi-automatic system, according to standardized parameters [20]. Participants were in a supine position with the head turned 45° contralateral to the side of scanning, and measurements were taken on the far wall of the common carotid artery (CCA). All measurements were made with an electronic caliper and were recorded as photocopies. The mean CIMT was calculated as the average of three readings of the IMT for both carotid arteries on a longitudinal scan at a point 10 mm proximal from the beginning of the dilation of each carotid artery bulb.

All statistical analyses were performed using SPSS software version 22 (IBM Corp., Armonk, NY, USA). Continuous variables were tested for normality using the Shapiro–Wilk test. Normally distributed data were presented as the mean ± standard deviation (SD), while non-normally distributed data were reported as the median with interquartile range (IQR).

For group comparisons, patients were stratified into tertiles of serum hepcidin (Group 1: ≤37.45 ng/mL, Group 2: 37.46–46.67 ng/mL, Group 3: >46.67 ng/mL). Continuous variables were compared using the Kruskal–Wallis test, and categorical variables using the Chi-square test. The Kruskal–Wallis test was used for group comparisons due to the non-normal distribution of several variables across hepcidin tertiles, as assessed by the Shapiro–Wilk test. ANOVA was not used to avoid assumptions of normality and homoscedasticity. Post hoc comparisons were performed using Dunn’s test with Bonferroni correction.

Associations between carotid intima–media thickness (CIMT) and clinical/biochemical parameters were examined using Spearman’s rank correlation coefficient (ρ). Scatterplots with fitted regression lines were generated to visually assess these associations.

To evaluate predictors of CIMT, linear regression analyses were performed. First, univariate regression was used to test each variable individually. Subsequently, a multivariate linear regression model was constructed to identify independent predictors of CIMT. Although non-parametric tests were used for group comparisons, the dependent variable (CIMT) in regression analysis was approximately normally distributed, as confirmed by residual plots and histogram analysis. Results are reported as beta coefficients (β), 95% confidence intervals (CI), and *p*-values. Statistical significance was set at *p* < 0.05. To evaluate the ability of hepcidin and other predictors to discriminate patients with elevated CIMT (≥0.9 mm), ROC curve analyses were performed, and the area under the curve (AUC) was calculated. Logistic regression models were used to derive predicted probabilities for multivariable ROC analysis.

A post hoc power analysis was conducted based on the observed correlation between serum hepcidin and CIMT (ρ = 0.788, *p* < 0.001). With 82 patients, the statistical power to detect this effect exceeded 99%, confirming adequacy for the primary analysis. Sample size estimations indicated that 13 patients would be sufficient to detect a large effect (ρ = 0.50), 41 patients for a moderate effect (ρ = 0.30), and approximately 95 patients for a small effect (ρ = 0.20) with 80% power at α = 0.05. These findings demonstrate that the study was adequately powered to detect clinically relevant associations between hepcidin and CIMT.

## 3. Results

A total of 82 patients undergoing PD were included in the study. Their baseline demographic and clinical characteristics are summarized in Table 1. The mean age was 55.85 ± 11.79 years, and 59.76% were male. The average duration of PD was 41.23 ± 15.38 months. Hypertension and diabetes mellitus were present in 48.78%, 57.32% of patients, respectively. The mean body mass index (BMI) was 23.85 ± 3.41 kg/m^2^, and 37.8% of patients reported a history of smoking. Erythropoiesis-stimulating agent (ESA) therapy was administered to 48.78% of patients, while 28.05% received intravenous (IV) iron supplementation.

Biochemical parameters are detailed in Table 2. Inflammatory and iron-related markers included a mean CRP level of 5.14 ± 2.35 mg/L and a mean serum hepcidin level of 42.12 ± 9.79 ng/mL. The average carotid intima–media thickness (CIMT) was 0.73 ± 0.15 mm.

To further assess the relationship between serum hepcidin levels and vascular remodeling, patients were stratified into tertiles according to serum hepcidin levels: Group 1 (≤37.45 ng/mL), Group 2 (37.46–46.67 ng/mL), and Group 3 (>46.67 ng/mL). The distribution of clinical and biochemical characteristics across tertiles is shown in Table 3.

**Figure 1:**
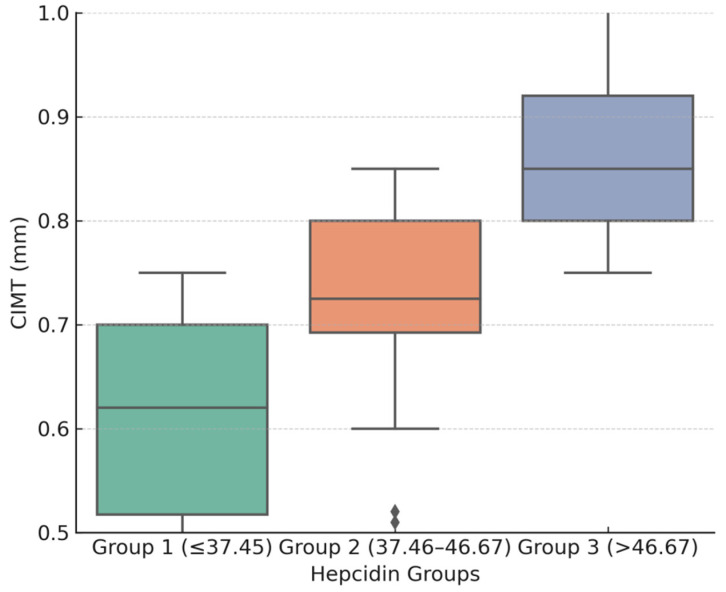
Mean CIMT values (±95% CI) across hepcidin tertiles, demonstrating a significant increase in the highest tertile. Group 1 is presented in green, Group 2 in pink, and Group 3 in purple.

The prevalence of hypertension, diabetes mellitus, and smoking increased progressively across tertiles, with the highest prevalence observed in Group 3 (all *p* < 0.001). Biochemical markers varied significantly: serum ferritin and CRP were markedly elevated in the higher tertiles. Notably, CIMT values increased stepwise across hepcidin tertiles, with Group 3 showing the greatest thickness compared with Groups 1 and 2 (*p* < 0.001). The trend is presented in Figure 1, which displays the mean CIMT values (±95% CI) across hepcidin tertiles. Interestingly, sex distribution differed significantly across tertiles, with the proportion of males decreasing progressively from Group 1 (78.6%) to Group 3 (31.2%) (*p* = 0.009). This inverse relationship between male sex and hepcidin levels may echo underlying gender-specific differences in iron metabolism or inflammation.

### 3.1. Correlation Analysis

Spearman correlation analysis was conducted to examine the associations between carotid intima–media thickness (CIMT) and various clinical and biochemical variables (Table 4). The results revealed strong positive correlations between CIMT and serum hepcidin (ρ = 0.788, *p* < 0.001), ferritin (ρ = 0.675, *p* < 0.001), transferrin saturation (TSAT) (ρ = 0.420, *p* < 0.001), and C-reactive protein (CRP) (ρ = 0.563, *p* < 0.001). Moderate positive correlations were observed with serum iron (ρ = 0.253, *p* = 0.002), hemoglobin (ρ = 0.224, *p* = 0.043), hematocrit (ρ = 0.216, *p* = 0.050), and PD duration (ρ = 0.250, *p* = 0.023).

Among categorical and treatment-related variables, CIMT showed a significant positive correlation with hypertension (ρ = 0.510, *p* < 0.001) and intravenous iron administration (ρ = 0.288, *p* = 0.009), while ESA therapy was negatively correlated (ρ = −0.221, *p* = 0.043). Gender was also negatively correlated (ρ = −0.288, *p* = 0.008).

### 3.2. Hepcidin and CIMT Relationship

**Figure 2:**
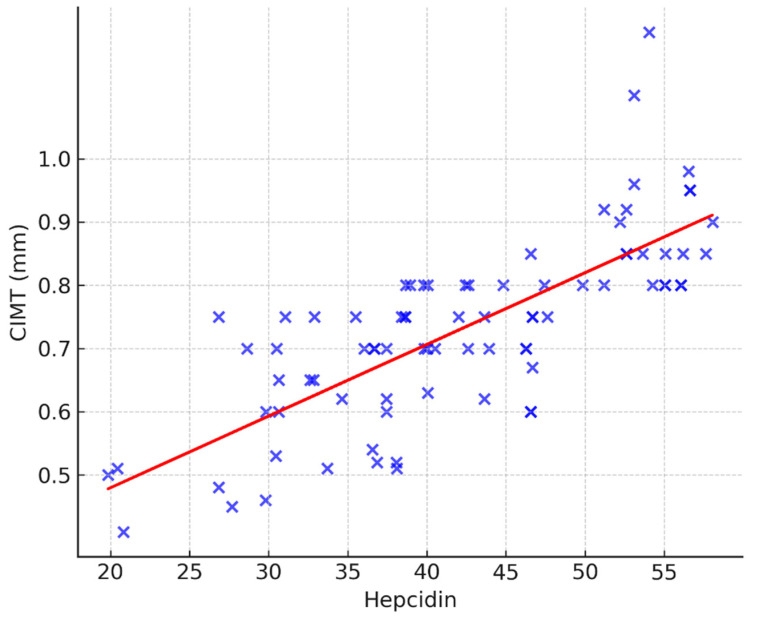
Scatterplot showing the association between serum hepcidin and carotid intima–media thickness (CIMT). A linear regression trend line is overlaid. The blue crosses depict the individual correlations between the two parameters, while the red line is the trendline, representing the correlation trend for the entire group.

The correlation of CIMT with hepcidin is additionally visualized in Figure 2. Higher serum hepcidin concentrations were consistently associated with greater CIMT values (ρ = 0.788, *p* < 0.001).

To further explore the predictors of CIMT as a continuous variable, univariate and multivariate linear regression analyses were conducted (Table 5).

In univariate linear regression analysis, gender (β = −0.0873, *p* = 0.0071), PD duration (β = 0.0028, *p* = 0.0075), hypertension (β = 0.1345, *p* < 0.001), DM (β = 0.1284, *p* < 0.001), smoking history (β = 0.1464, *p* < 0.001), IV iron therapy (β = −0.094, *p* = 0.0079), Hct (β = 0.1275, *p* = 0.041), Hgb (β = 0.0021, *p* = 0.043), TSAT (β = 0.0023, *p* = 0.005), ferritin (β = 0.0009, *p* < 0.001), CRP (β = 0.0362, *p* < 0.001), and hepcidin (β = 0.0057, *p* = 0.012) were significant predictors of CIMT.

In the multivariate model, PD duration, hypertension, and hepcidin remained independent predictors of CIMT, whereas other covariates lost statistical significance.

### 3.3. ROC Curve Analysis

**Figure 3:**
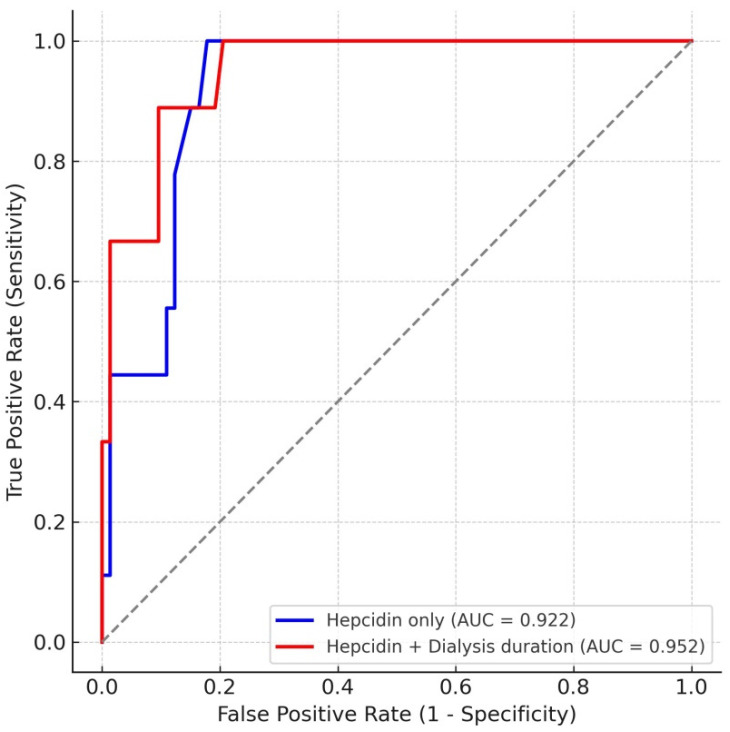
ROC curves for prediction of high CIMT (≥0.9 mm) using serum hepcidin alone and in combination with dialysis duration. The dashed line outlines the area under the curve (AUC).

To further evaluate the diagnostic utility of hepcidin as a predictor of subclinical atherosclerosis, we performed receiver operating characteristic (ROC) curve analysis using CIMT ≥ 0.9 mm as the threshold for high-risk patients (Figure 3). Hepcidin alone demonstrated excellent discriminative performance, with an AUC of 0.922 (95% CI 0.843–0.976). The optimal cutoff value was 51.2 ng/mL, which provided 100% sensitivity and 82% specificity. When dialysis duration was incorporated into the model, the predictive accuracy further improved (AUC = 0.952). In multivariable logistic regression, both hepcidin (*p* = 0.010) and dialysis duration (*p* = 0.023) were independent predictors of high CIMT. These findings indicate that hepcidin is a robust biomarker for subclinical atherosclerosis in PD patients, and that dialysis duration adds complementary prognostic value.

## 4. Discussion

In this study, we demonstrated that serum hepcidin levels are strongly associated with subclinical atherosclerosis, as assessed by carotid intima–media thickness (CIMT), in patients undergoing peritoneal dialysis (PD). Hepcidin showed a robust correlation with CIMT and remained an independent predictor in multivariate linear regression analysis, together with dialysis duration. These findings support the role of hepcidin not only as a biomarker of iron metabolism but also as a potential mediator of vascular injury in PD patients.

It has been more than 40 years since iron was first implicated in the context of atherosclerosis [21]. Since then, numerous in vitro [22] and in vivo [23,24] studies have been conducted to investigate this relationship; however, they have generated contradicting results and are still being debated in the literature.

Identification of hepcidin in 2001 and clarification of the mechanisms of its actions established this hormone as the principal regulator of systemic iron metabolism [25,26]. This small peptide hormone expresses its regulatory function by inhibiting the uptake of iron in the gut and preventing the release of stored iron from macrophages [27]. Secretion of hepcidin is upregulated by increased iron stores and inflammation, whereas it is reduced in response to iron deprivation, hypoxia, and enhanced erythropoiesis [7].

Multiple studies have highlighted the impact of hepcidin on CV risk in different patient populations, including those with CKD [28,29,30]. In a study in patients receiving hemodialysis, conducted by Anupama et al., hepcidin levels were linked to CV mortality, leading the authors to conclude that hepcidin may be a reliable predictor of CV outcomes [31]. Erdogan et al. found that hepcidin-25 was positively correlated with age, CRP, ferritin, and CIMT in PD patients, suggesting that inflammation and iron overload may converge in promoting vascular changes [32]. Similarly, Yayar et al. demonstrated that higher hepcidin levels predicted increased CIMT and were associated with cardiovascular mortality in hemodialysis patients [33]. On the other hand, Zhong et al. have found a significant correlation between hepcidin, all-cause and infection-related mortality in a large group of PD patients, but not between hepcidin and CV mortality [34]. They have implicated that hepcidin has a significant role as a host-defense mediator during the early phases of infection [35], but emphasized that it may damage cellular defense against certain intracellular infections by accelerating retention of iron in macrophages [36], potentially compromising their ability to kill pathogens and leading to undesirable outcomes.

The biological plausibility of the relationship between hepcidin and vascular remodeling is grounded in the pro-inflammatory and iron-sequestering role of hepcidin, particularly in macrophages. By limiting iron efflux through ferroportin degradation, hepcidin promotes intracellular iron retention, contributing to oxidative stress and foam cell formation—a key step in atherogenesis [37].

The duration of PD treatment is the other independent predictor of CIMT in our patients. Although life-saving, PD often promotes inflammation, a key factor driving atherosclerosis [38]. The factors promoting inflammation in these patients are complex. The bio-incompatible PD fluids induce local inflammation of the peritoneal cavity, leading to membrane injury and dysfunction [39]. High glucose concentrations in these fluids, which are necessary for ultrafiltration, contribute to undesirable metabolic effects [39]. Despite using glucose alternatives and making efforts to reduce glucose levels and advanced glycation end products, the pro-inflammatory consequences of PD therapy remain significant [40]. The disruption of the peritoneal membrane integrity with a PD catheter and biofilm formation within the catheter lumen due to skin bacteria also contributes to local peritoneal inflammation [41]. These patients also have high levels of pro-inflammatory cytokines driven by the uremic milieu and impaired clearance. Some data show that the level of pro-inflammatory cytokines increases with the duration of PD treatment [42], and can be explained by the decline in residual renal function and peritoneal ultrafiltration over time due to endothelial changes, peritoneal angiogenesis, inflammatory response, and oxidative stress [43]. This promotes systemic inflammation, accelerating atherosclerosis and resulting in plaque formation [44].

We have analyzed the relationship between hepcidin and CIMT further by stratifying the patients into groups based on hepcidin levels. Consistent increases in iron metabolism markers, including serum iron, transferrin saturation (TSAT) and ferritin levels, were observed across the hepcidin tertiles, depicting the regulatory role that hepcidin plays in iron homeostasis. However, the fact that hepcidin levels correlated with PD duration may suggest that long-term PD treatment contributes to vascular changes through mechanisms related to iron dysregulation and chronic inflammation. Interestingly, we observed a significant gender imbalance across serum hepcidin tertiles, with lower proportions of male patients in the higher hepcidin groups. This distribution may have influenced some of the biochemical parameters, as sex differences in hepcidin regulation are well documented, with females generally exhibiting higher hepcidin levels due to lower iron stores and hormonal regulation [45]. Therefore, gender should be considered as a potential confounder in interpreting the hepcidin–CIMT relationship, and future studies should control for sex-specific effects more rigorously.

CIMT itself has been widely validated as a marker of early atherosclerosis and a predictor of cardiovascular morbidity and mortality in dialysis patients [46]. Our finding that CIMT increased progressively across hepcidin tertiles strengthens the argument for a direct hepcidin-vascular axis. The association may be explained by multiple mechanisms: (1) iron sequestration and macrophage iron overload promoted by hepcidin, leading to oxidative stress and vascular damage; (2) pro-inflammatory effects, as suggested by correlations with CRP; and (3) an indirect link with anemia management, since patients in higher tertiles were more frequently treated with intravenous iron and ESA therapy.

Interestingly, in our multivariate analysis, traditional cardiovascular risk factors such as age and diabetes did not remain significant predictors of CIMT. This suggests that non-traditional risk factors, including iron homeostasis and chronic inflammation, may play a more dominant role in vascular pathology among PD patients. Similar findings have been reported in hemodialysis cohorts, where hepcidin and age were independent determinants of CIMT [37].

Our ROC analysis demonstrated that serum hepcidin alone provided excellent discriminative power for identifying patients with high CIMT (AUC = 0.922), and the predictive performance was further enhanced when dialysis duration was incorporated into the model (AUC = 0.952). These findings support the potential role of hepcidin as a biomarker for subclinical atherosclerosis in PD patients, with additive value from dialysis-related factors.

Similar observations have been made in hemodialysis cohorts. Xu et al. reported that elevated serum hepcidin was independently associated with cardiovascular disease in maintenance HD patients, and ROC analysis identified hepcidin as a useful diagnostic marker [47].

In another study, El Sewefy et al. showed that hepcidin levels ≥ 280 ng/mL had an 83% accuracy in discriminating ESA-resistant from -responsive HD patients, reinforcing its diagnostic potential [48]. Furthermore, van der Weerd et al. demonstrated that hepcidin-25 was related to fatal and non-fatal cardiovascular events in HD patients, even after adjustment for inflammation [49].

From a clinical point of view, our findings raise the possibility of hepcidin as a therapeutic target or a biomarker to guide iron therapy in PD patients. Elevated hepcidin may signal heightened vascular risk, suggesting the need for careful balancing of iron supplementation and ESA use. Prospective studies are warranted to confirm whether modulation of hepcidin levels can alter cardiovascular outcomes in this vulnerable group.

### Strengths and Limitations

The major strength of this study is the comprehensive evaluation of iron metabolism, inflammation, and traditional risk factors in relation to CIMT. Our cohort size (n = 82) provided adequate power (>99%) to detect large associations between hepcidin and CIMT.

Our study has several limitations. It is cross-sectional in nature, preventing causal inference. Although our analysis was well powered for moderate-to-large effect sizes, it may still be underpowered to detect small associations. Additionally, the absence of a control group limits the generalizability of our findings. Comparative analyses with healthy controls or other CKD populations would further strengthen the findings.

Furthermore, residual confounding by unmeasured factors such as dietary iron intake or genetic polymorphisms in iron metabolism cannot be excluded. Additionally, the uneven gender distribution across hepcidin tertiles may have introduced confounding effects, given known sex-based differences in iron metabolism and cardiovascular risk profiles. Although gender was included in the multivariate model, residual confounding cannot be excluded. FGF23 was not included in our analysis due to incomplete data, despite accumulating evidence indicating that FGF23, together with phosphate metabolism, plays an important role in vascular remodeling in CKD and dialysis patients.

Vascular calcification in dialysis patients represents a multifactorial process caused by chronic inflammation, oxidative stress, and mineral metabolism disorders, within which hepcidin likely acts as one contributor rather than an isolated driver. Moreover, peritoneal dialysis involves chronic glucose exposure that may further promote endothelial dysfunction and vascular injury. Therefore, while our findings support the potential role of hepcidin as an early marker of atherosclerotic remodeling, it should be interpreted within the broader context of systemic metabolic and inflammatory stressors.

Prospective cohort studies and mechanistic investigations are warranted to confirm whether interventions targeting hepcidin (e.g., hepcidin antagonists, individualized iron therapy) could mitigate cardiovascular risk.

## 5. Conclusions

In summary, our study recognizes serum hepcidin as an independent predictor of subclinical atherosclerosis, measured by carotid intima–media thickness, in peritoneal dialysis patients. These findings suggest a possible role for hepcidin in dialysis-related vascular remodeling and highlight its potential as a biomarker for cardiovascular risk stratification. Future longitudinal studies are warranted to determine causality and evaluate therapeutic implications.

## Figures and Tables

**Table 1:** Patient demographics and clinical characteristics.

Variable	
Age (years)	55.85 ± 11.79
Gender (male) %	59.76%
PD duration (months)	41.23 ± 15.38
Hypertension (%)	48.78%
DM (%)	57.32%
Smoking history%	37.80%
BMI (kg/m^2^)	23.85 ± 3.41
Medications	
ESA	48.78%
IV iron treatment	28.05%

Abbreviations: PD, peritoneal dialysis; DM, diabetes mellitus; BMI, body mass index; ESA, erythropoiesis-stimulating agents; IV, intravenous.

**Table 2:** Biochemical characteristics of the group.

Variable	Mean ± SD
Total cholesterol (mmol/L)	5.91 ± 1.36
LDL-C (mmol/L)	3.73 ± 1.13
HDL-C (mmol/L)	1.12 ± 0.3
Triglycerides (mmol/L)	2.01 ± 0.84
Ca (mmol/L)	2.19 ± 0.22
P (mmol/L)	1.44 ± 0.42
Ca X P	3.16 ± 0.94
Hct (L/L)	0.35 ± 0.06
Hgb (g/L)	111.61 ± 13.17
Fe (µmol/L)	11.87 ± 1.9
TIBC	43.69 ± 7.68
TSAT (%)	28.5 ± 11.24
Ferritin (µg/L)	281.06 ± 109.62
CRP (mg/L)	5.14 ± 2.35
Hepcidin (ng/mL)	42.12 ± 9.79
CIMTav (mm)	0.73± 0.15

Abbreviations: LDL-C, low-density lipoprotein cholesterol; HDL-C, high-density lipoprotein cholesterol. Ca, serum calcium; P, serum phosphorus; Hct, hematocrit; Hgb, hemoglobin; Fe, serum iron; TIBC, total iron binding capacity; TSAT, transferrin saturation; CRP, C-reactive protein; CIMTav, carotid artery intima–media thickness average.

**Table 3:** Patients’ baseline characteristics stratified by tertiles of serum hepcidin levels.

Variable	Group 1 (≤37.45)	Group 2 (37.46–46.67)	Group 3 (>46.67)	*p*-Value
Age (years)	56.07 ± 15.45	55.29 ± 10.65	56.23 ± 8.35	0.99
Gender (male)	78.6%	68.1%	31.2%	0.009
PD duration (months)	32.50 ± 15.40	39.68 ± 15.13	47.77 ± 15.36	0.0025
Hypertension (%)	29.4%	65.7%	81.2%	<0.001
DM (%)	18.1%	43%	88.2%	<0.001
Smoking history (%)	7.1%	29.2%	80.8%	<0.001
BMI (kg/m^2^)	23.80 ± 2.86	23.59 ± 3.18	24.17 ± 4.21	0.84
ESA	21.4%	71.4%	61.5%	0.063
IV iron treatment	39.3%	92.9%	84.6%	0.0088
Total cholesterol (mmol/L)	5.85 ± 1.05	6.38 ± 1.40	5.48 ± 1.49	0.17
LDL-C (mmol/L)	3.88 ± 1.00	3.47 ± 1.09	3.84 ± 1.30	0.24
HDL-C (mmol/L)	1.16 ± 0.26	1.13 ± 0.31	1.05 ± 0.33	0.15
Triglycerides (mmol/L)	2.00 ± 0.92	1.86 ± 0.74	2.18 ± 0.86	0.43
Ca (mmol/L)	2.19 ± 0.2	2.27 ± 0.24	2.11 ± 0.18	0.03
P (mmol/L)	1.4 ± 0.37	1.43 ± 0.32	1.49 ± 0.55	0.79
Ca X P	3.07 ± 0.86	3.25 ± 0.79	3.15 ± 1.16	0.64
Hgb (g/L)	105.24 ± 9.89	113.42 ± 11.36	118.0 ± 11.41	0.002
Hct (L/L)	0.32 ± 0.05	0.37 ± 0.06	0.36 ± 0.07	0.0013
Fe (µmol/L)	11.06 ± 1.4	12.3 ± 1.16	12.29 ± 2.64	0.016
Ferritin (µg/L)	196.50 ± 82.06	262.25 ± 72.48	392.37 ± 68.53	<0.001
TIBC (µmol/L)	43.0 ± 8.5	44.1 ± 7.3	43.8 ± 7.3	0.9184
TSAT (%)	25.7 ± 10.6	29.0 ± 11.2	31.0 ± 11.1	0.0433
CRP (mg/L)	3.46 ± 1.69	5.09 ± 2.29	7.02 ± 1.64	<0.001
Hepcidin (ng/mL)	31.48 ± 5.15	42.18 ± 3.16	53.5 ± 3.1	<0.001
CIMTav (mm)	0.60 ± 0.1	0.71 ± 0.05	0.88 ± 0.1	<0.001

Abbreviations: PD, peritoneal dialysis; DM, diabetes mellitus; BMI, body mass index; ESA, erythropoiesis-stimulating agents; IV, intravenous; LDL-C, low-density lipoprotein cholesterol; HDL-C, high-density lipoprotein cholesterol. Ca, serum calcium; P, serum phosphorus; Hgb, hemoglobin; Hct, hematocrit; Fe, serum iron; TIBC, total iron binding capacity; TSAT, transferrin saturation; CRP, C-reactive protein; CIMTav, carotid artery intima–media thickness average.

**Table 4:** Correlation analysis of variables with CIMT.

Variable	Correlation Coefficient (ρ)	*p*-Value
Age	0.181	0.103
Gender	−0.288	0.008 **
PD duration	0.25	0.023 *
Hypertension	0.51	<0.001 ***
DM	0.059	<0.001 ***
Smoking history	0.0639	<0.001 ***
BMI	0.127	0.2579
ESA	−0.221	0.043 *
IV iron treatment	0.288	0.009 **
Total cholesterol	−0.207	0.062
LDL-C	−0.191	0.085
HDL-C	−0.14	0.211
Triglycerides	0.075	0.502
Ca	−0.136	0.224
P	0.076	0.499
Ca x P	0.092	0.4109
Hct	0.216	0.05 *
Hgb	0.224	0.043 *
Fe	0.253	0.002 **
TSAT	0.42	<0.001 ***
Ferritin	0.675	<0.001 ***
CRP	0.563	<0.001 ***
Hepcidin	0.788	<0.001

* *p*  <  0.05, ** *p*  <  0.01, and *** *p*  <  0.001.

**Table 5:** Linear Regression Analysis of CIMT Predictors.

Variable	Univariate Linear Regression			Multivariable Linear Regression	
	Beta (95% CI)	*p*-Value	R^2^	Beta (95% CI)	*p*-Value
Gender	−0.0873 (−0.1502–−0.0245)	0.0071	0.087	−0.0023 (−0.0496–0.045)	0.9228
PD duration	0.0028 (0.0008–0.0048)	0.0075	0.086	0.0018 (0.0004–0.0032)	0.0153
Hypertension	0.1345 (0.0766–0.1924)	<0.001	0.211	0.0553 (0.0076–0.103)	0.0237
DM	0.1284 (0.0705–0.1862)	<0.001	0.196	0.0322 (−0.0231–0.0875)	0.2499
Smoking history	0.1464 (0.0884–0.2044)	<0.001	0.24	−0.0189 (−0.0786–0.0407)	0.5285
ESA	−0.0464 (−0.1101–0.0173)	0.1514	0.026	0.02 (−0.0274–0.0674)	0.4034
IV iron therapy	−0.094 (−0.1627–−0.0253)	0.0079	0.085	−0.0127 (−0.0653–0.04)	0.6326
Hct	0.1275 (0.022–0.233)	0.041	0.051	0.1042 (0.021–0.187)	0.062
Hgb	0.0021 (0.0005–0.0037)	0.043	0.049	0.0012 (−0.0001–0.0025)	0.071
TSAT	0.0023 (0.0007–0.0039)	0.005	0.061	0.0020 (0.0001 to 0.0039)	0.052
Ferritin	0.0009 (0.0006–0.0011)	<0.001	0.424	0.0002 (−0.0001–0.0006)	0.1222
CRP	0.0362 (0.025–0.0474)	<0.001	0.341	0.0095 (−0.0021–0.0211)	0.1062
Hepcidin	0.0113 (0.0092–0.0135)	<0.001	0.579	0.0057 (0.0013–0.0101)	0.0122

## Data Availability

The data that support the findings of this study are available from the corresponding author upon reasonable request.
